# The dopamine β-hydroxylase -1021C/T polymorphism is associated with the risk of Alzheimer's disease in the Epistasis Project

**DOI:** 10.1186/1471-2350-11-162

**Published:** 2010-11-11

**Authors:** Onofre Combarros, Donald R Warden, Naomi Hammond, Mario Cortina-Borja, Olivia Belbin, Michael G Lehmann, Gordon K Wilcock, Kristelle Brown, Patrick G Kehoe, Rachel Barber, Eliecer Coto, Victoria Alvarez, Panos Deloukas, Rhian Gwilliam, Reinhard Heun, Heike Kölsch, Ignacio Mateo, Abderrahim Oulhaj, Alejandro Arias-Vásquez, Maaike Schuur, Yurii S Aulchenko, M Arfan Ikram, Monique M Breteler, Cornelia M van Duijn, Kevin Morgan, A David Smith, Donald J Lehmann

**Affiliations:** 1 Neurology Service and Centro de Investigación Biomédica en Red sobre Enfermedades Neurodegenerativas (CIBERNED), Marqués de Valdecilla University Hospital (University of Cantabria), 39008 Santander, Spain; 2 Oxford Project to Investigate Memory and Ageing (OPTIMA), University Department of Physiology, Anatomy and Genetics, South Parks Road, Oxford OX1 3QX, UK; 3 The Wellcome Trust Sanger Institute, Hinxton, Cambridge CB10 1SA, UK; 4 Centre for Paediatric Epidemiology and Biostatistics, Institute of Child Health, University College London, 30 Guilford Street, London WC1N 1EH, UK; 5 School of Molecular Medical Sciences, Institute of Genetics, University of Nottingham, Queens Medical Centre, Nottingham NG7 2UH, UK; 6 Nuffield Department of Medicine, University of Oxford, Level 4, John Radcliffe Hospital, Oxford OX3 9DU, UK; 7 Dementia Research Group, Institute of Clinical Neurosciences, University of Bristol, Frenchay Hospital, Frenchay Bristol, BS16 1LE, UK; 8 Genética Molecular, Hospital Central de Asturias, Oviedo, Spain; 9 Department of Psychiatry, University of Bonn, Bonn, Germany; 10 Royal Derby Hospital, Uttoxeter Road, Derby DE22 3NE, UK; 11 OPTIMA, Nuffield Department of Medicine, University of Oxford, Level 4, John Radcliffe Hospital, Oxford OX3 9DU, UK; 12 Department of Epidemiology, Erasmus MC University Medical Center, Rotterdam, the Netherlands; 13 Department of Psychiatry, Donders Institute for Brain, Cognition and Behavior & Department of Human Genetics, Radboud University Nijmegen, Medical Centre, P.O. Box 9101, HP 966, Nijmegen 6500 HB, The Netherlands; 14 Department of Neurology, Erasmus MC University Medical Center, Rotterdam, the Netherlands

## Abstract

**Background:**

The loss of noradrenergic neurones of the locus coeruleus is a major feature of Alzheimer's disease (AD). Dopamine β-hydroxylase (DBH) catalyses the conversion of dopamine to noradrenaline. Interactions have been reported between the low-activity -1021T allele (rs1611115) of *DBH *and polymorphisms of the pro-inflammatory cytokine genes, *IL1A *and *IL6*, contributing to the risk of AD. We therefore examined the associations with AD of the *DBH *-1021T allele and of the above interactions in the Epistasis Project, with 1757 cases of AD and 6294 elderly controls.

**Methods:**

We genotyped eight single nucleotide polymorphisms (SNPs) in the three genes, *DBH*, *IL1A *and *IL6*. We used logistic regression models and synergy factor analysis to examine potential interactions and associations with AD.

**Results:**

We found that the presence of the -1021T allele was associated with AD: odds ratio = 1.2 (95% confidence interval: 1.06-1.4, *p *= 0.005). This association was nearly restricted to men < 75 years old: odds ratio = 2.2 (1.4-3.3, 0.0004). We also found an interaction between the presence of *DBH *-1021T and the -889TT genotype (rs1800587) of *IL1A*: synergy factor = 1.9 (1.2-3.1, 0.005). All these results were consistent between North Europe and North Spain.

**Conclusions:**

Extensive, previous evidence (reviewed here) indicates an important role for noradrenaline in the control of inflammation in the brain. Thus, the -1021T allele with presumed low activity may be associated with misregulation of inflammation, which could contribute to the onset of AD. We suggest that such misregulation is the predominant mechanism of the association we report here.

## Background

### Noradrenergic neurones in Alzheimer's disease

The loss of noradrenergic neurones of the locus coeruleus is a striking feature of sporadic Alzheimer's disease (AD). A gradual, moderate loss is found with ageing in healthy people [1-3], but a more dramatic loss is seen in AD. A meta-analysis [4] showed similarly high losses of noradrenergic neurones (24 studies) as of cholinergic neurones (33 studies), with losses four times greater than those of dopaminergic cells in AD. Noradrenergic neurones project from the brainstem to innervate wide areas of the forebrain [5]. Levels of noradrenaline (NA, norepinephrine) in target regions have also sometimes been reported lowered in ageing [6,7], e.g. in the hippocampus and hypothalamus. They have generally been found to be further reduced in AD [8-13], e.g. in the hippocampus, hypothalamus, caudate nucleus, putamen and neocortex, although not in one small study [14]. Both the loss of noradrenergic neurones [15] and that of NA in target regions [8,13,16] have been correlated with the severity of the disease. Changes in the noradrenergic system in AD are reviewed in Hermann et al 2004 [17].

### Dopamine β-hydroxylase -1021C/T

Dopamine β-hydroxylase (DBH) catalyses the conversion of dopamine to NA. Its activity is also reduced in postmortem hippocampus and neocortex in AD [18,19], without correlating with the loss of noradrenergic neurones [19]. Variation in DBH activity both in serum and in CSF has been reported to be over 80% heritable [20]. The single nucleotide polymorphism (SNP), -1021C/T (rs1611115), has been identified as the main predictor of DBH activity in plasma [21,22]. It is responsible for ~30% to ~50% of the considerable variation in such activity between people, as replicated in several different populations [21,23-27]. The -1021T allele contributes to greatly lowered DBH activity through codominant inheritance [21]. In view therefore of the chronic inflammation seen in the AD brain [28,29] and of the anti-inflammatory role of NA [30], Mateo et al 2006 [31] investigated interactions between the -1021T allele and SNPs of the regulatory regions of the pro-inflammatory cytokine genes, *IL1A *and *IL6*. They reported interactions between *DBH *-1021TT and both *IL1A *-889T (rs1800587) and *IL6 -*174GG (rs1800795). In the Epistasis Project, we recently confirmed [32] reported interactions between the inflammation-related cytokine genes, *IL6 *and *IL10*, that contribute to the development of AD. We therefore now decided also to examine the interactions between *DBH *and both *IL1A *and *IL6 *in the Epistasis Project, with 1757 cases of AD and 6294 controls. In view of the age and sex differences that have been reported in brain inflammation in the elderly [33], and of the relevant influence of sex steroids [34], we also examined possible interactions of *DBH *with age and sex. We found an association of the low-activity *DBH *-1021T allele with the risk of AD.

## Methods

### Study population

The Epistasis Project aims primarily to replicate interactions that have been reported to affect the risk of AD. Sample-sets were drawn from narrow geographical regions with relatively homogeneous, Caucasian populations, by seven AD research groups: Bonn, Bristol, Nottingham, OPTIMA (Oxford), Oviedo, Rotterdam and Santander. Sample characteristics by geographical region are given in Additional file 1: Table S1. All AD cases were diagnosed "definite" or "probable" by CERAD or NINCDS-ADRDA criteria. AD cases were sporadic, i.e. possible autosomal dominant cases were excluded, based on family history. The median ages (interquartile ranges) of AD cases were 79.0 (73.0-85.2) and of controls were 76.9 (71.3-83.0). Fuller details of our sample-sets are given elsewhere [32]. Ethical approval was obtained by each of the participating groups (Additional file 1: Table S2).

### Genotyping

Blood samples were taken after written informed consent had been obtained from the subjects or their representatives. Genotyping for the six centres other than Rotterdam (below) was performed at the Wellcome Trust Sanger Institute, using the iPLEX Gold assay (Sequenom Inc.). Whole genome amplified DNA was used for 82% of samples; genomic DNA was used for the 18% of samples that were not suitable for whole genome amplification. A Sequenom iPLEX, designed for quality control purposes, was used to assess genotype concordance between genomic and whole genome amplified DNA for 168 individuals. Assays for all SNPs were designed using the eXTEND suite and MassARRAY Assay Design software version 3.1 (Sequenom Inc.). Samples were amplified in multiplexed PCR reactions before allele specific extension. Allelic discrimination was obtained by analysis with a MassARRAY Analyzer Compact mass spectrometer. Genotypes were automatically assigned and manually confirmed using MassArray TyperAnalyzer software version 4.0 (Sequenom Inc.). Gender markers were included in all iPLEX assays as a quality control metric for confirmation of plate/sample identity. Genotyping of *DBH *intron 10 A/G (rs1611131) and *IL6 *intron 2 A/G (rs2069837) was carried out using KASPar technology by KBioscience http://www.kbioscience.co.uk. No SNPs were imputed.

Genotyping in the Rotterdam cohort was done on Version 3 Illumina-Infinium-II HumanHap550 SNP array (Illumina, San Diego, USA) and additionally, SNPs were imputed using MACH software http://www.sph.umich.edu/csg/abecasis/MACH/ with HapMap CEU Release 22 as a reference [35]. The reliability of imputation was estimated for each imputed SNP with the ratio of expected and observed dosage variance (O/E ratio). Only samples with high-quality extracted DNA were genotyped; 5974 were available with good quality genotyping data; 5502 of these had reliable phenotypes. For this study, *DBH *exon 3 Ala197Thr (rs5320), *IL1A *exon 5 Ala114Ser (rs17561) and *IL6 *intron 2 A/G (rs2069837) were genotyped, and the other SNPs (Table 1) were imputed.

**Table 1:** Studied SNPs

Gene	SNP		Minor allele frequency in controls			Linkage disequilibrium in controls				
			
			North Europe	North Spain	Difference (*p*)	With	North Europe		North Spain	
							
							*D'*	*r^2^*	*D'*	*r^2^*
*DBH*	rs1611115	-1021C/T	20.7% (T)	19.7% (T)	0.47	rs5320	0.994	0.015	0.994	0.017
	rs 5320	Exon 3 Ala197Thr	5.3% (A, Thr)	6.0% (A, Thr)	0.38	rs1611131	0.257	0.002	0.393	0.003
	rs1611131	Intron 10 A/G	29.4% (G)	24.5% (G)	**0.002**	rs1611115	0.295	0.055	0.250	0.047

*IL1A*	rs1800587	-889C/T	29.2% (T)	25.4% (T)	**0.01**	rs17561	0.999	0.994	0.989	0.971
	rs17561	Exon 5 Ala114Ser	29.2% (T, Ser)	25.6% (T, Ser)	**0.02**	rs3783550	0.997	0.185	0.999	0.145
	rs3783550	Intron 6 A/C	31.2% (C)	29.8% (C)	0.39	rs1800587	0.994	0.185	0.999	0.144

*IL6*	rs1800795	-174G/C	41.1% (C)	32.8% (C)	**4 × 10 ^-7^**	rs2069837	0.999	0.055	0.998	0.049
	rs2069837	Intron 2 A/G	7.3% (G)	9.3% (G)	**0.03**					

SNP = single nucleotide polymorphism, *DBH *= dopamine β-hydroxylase, *IL1A *= interleukin-lα, *IL6 *= interleukin-6, *D' *= ratio of observed linkage disequilibrium to maximum possible linkage disequilibrium, *r^2 ^*= correlation coefficient.

Results in bold are significant at *p *< 0.05.

### Statistical analysis

We assessed associations with logistic regression models, controlling for age, gender, study centre and the ε4 allele of apolipoprotein E (*APOE*ε4), using R Version 2.10.1 (R Foundation for Statistical Computing, Vienna, Austria). The adjusted synergy factors [36] were derived from the interaction terms in those models. Since both -1021TT and -1021TC are associated with reduced plasma DBH activity, although the former more so than the latter, we combined the two genotypes in all analyses, i.e. using a model that assumes that the -1021T allele is dominant. For reasons of power, it is usual to use minor-allele-dominant models in interaction analyses, even where a codominant model might produce a better fit. This is the almost invariable practice with the *APOE*ε4 allele.

Heterogeneity among centres was controlled thus. We first fitted a model including random effect terms by centre, which accounts for correlated (clustered) observations within populations while avoiding estimating extra parameters in the regression models. We then fitted centre as a fixed effect term with six contrasts. We compared the goodness of fit of both approaches using Akaike's Information Criterion, which penalises the model's likelihood by a function of the number of parameters in the model. We found that the model with fixed effect terms by centre was preferable and used it to control for different frequencies between populations. Overdispersion was controlled by fitting generalized linear models with a quasi-binomial family with logit link.

Where the overall synergy factor was significant at p < 0.05, the seven individual centres and the two geographical regions, North Europe and North Spain, were also examined. In view of the genetic differences found between North and South Europe in previous studies [37-39] and in the Epistasis Project (Table 1, Additional file 1: Table S1, and [40]), we included separate analyses for North Europe and North Spain. North Europe here comprises Bonn, Bristol, Nottingham, Oxford and Rotterdam; North Spain comprises Oviedo and Santander.

Power calculations were based on the observed synergy factor values. A Cox proportional hazards model, with a frailty term to account for centre differences, controlling also for sex and *APOE*4, was fitted to see whether the *DBH *-1021T allele was associated with the onset age of AD, after confirming the assumption of proportional hazards. Comparisons of allelic frequencies between North Spain and North Europe were by Fisher's exact test. Linkage disequilibrium data were estimated using the R genetics library http://cran.r-project.org/web/packages/genetics/index.html. All tests of significance and power calculations were two-sided.

## Results

### The data

Table 1 shows the allelic frequencies and patterns of linkage disequilibrium of the eight studied SNPs in controls. There were differences between North Europe and North Spain in allelic frequencies of five SNPs. *IL1A *-889C/T and exon 5 Ala114Ser were in almost 100% linkage disequilibrium. Genotype distributions of the eight SNPs in AD and controls from each of the seven centres are shown in Additional file 1: Table S3; allelic frequencies by country are given in Additional file 1: Table S4. Hardy-Weinberg analysis was performed for both cases and controls, both in the Rotterdam samples and in the samples from the other six centres, which were genotyped by the Sanger Institute. In three of these 32 analyses, the samples were not in Hardy-Weinberg equilibrium, compared with two as would be expected by chance. Those three sample-sets were all AD cases from the six centres: *IL1A *-889C/T (*p *= 0.03) and intron 6 A/C (*p *= 0.004), and *IL6 *-174G/C (*p *= 0.02). Since another SNP, Arg535Cys in exon 11 of *DBH *(rs6271), has also been reported to influence plasma DBH activity [23,24], although much less so than -1021C/T, we performed preliminary analysis of that SNP on data from six centres, i.e. excluding Rotterdam.

### Associations of *DBH* -1021TT+TC with AD

*DBH *-1021TT+TC versus CC was associated with AD overall: odds ratio = 1.2 (95% confidence interval: 1.06-1.4, *p *= 0.005). There were interactions with sex and age (Table 2). The interaction with sex was significant overall and in North Europe, while that with age was significant overall and in North Spain. In view of those interactions, we stratified our analyses by age and by sex. Those stratified analyses established that the observed association of *DBH *-1021TT+TC with AD in the population was due to an association nearly restricted to men < 75 years old: odds ratio = 2.2 (1.4-3.3, 0.0004) (Table 3). Similar results were obtained in North Europe and North Spain (Table 4). The *DBH *-1021T allele was not associated with onset age of AD.

**Table 2:** Interactions of *DBH *-1021TT+TC versus CC with sex and age in AD risk

Interaction	Dataset	Numbers		Power*	Adjusted^† ^synergy factor (95% CI, *p*)
				
		Controls	AD		
With sex	All	6201	1611	88%	**1.4 (1.1-1.9, 0.01)**
	North Europe	5708	1109	78%	**1.6 (1.1-2.1, 0.006)**
	North Spain	493	502	32%	1.3 (0.7-2.5, 0.4)

With age	All	6200	1611	85%	**1.4 (1.04-1.9, 0.03)**
(± 75 years)	North Europe	5708	1109	73%	1.3 (0.9-1.8, 0.2)
	North Spain	492	502	32%	**2.1 (1.1-3.9, 0.02)**

*DBH *= dopamine β-hydroxylase, AD = Alzheimer's disease, CI = confidence interval.

* power to detect a synergy factor of 1.4, as in the overall dataset, at *p *< 0.05.

^† ^All analyses controlled for centre, age, sex and genotype of apolipoprotein E ε4.

Results in bold are significant at *p *< 0.05.

**Table 3:** Odds ratios of AD for *DBH *-1021TT+TC vs CC, stratified by sex and by age

Subset	Adjusted* odds ratios of AD (95% CI, *p*)
Men	**1.6 (1.2-2.0, 0.0002)**
Women	1.05 (0.9-1.2, 0.60)
All < 75 years	**1.6 (1.2-2.2, 0.001)**
All > 75 years	1.06 (0.9-1.3, 0.47)

Men < 75 years	**2.2 (1.4-3.3, 0.0004)**
Men > 75 years	1.35 (0.98-1.8, 0.06)
Women < 75 years	1.3 (0.9-1.9, 0.24)
Women > 75 years	0.95 (0.8-1.2, 0.66)

AD = Alzheimer's disease, *DBH *= dopamine β-hydroxylase, CI = confidence interval.

* All analyses controlled for centre, age, sex and genotype of apolipoprotein E ε4.

Results in bold are significant at *p *< 0.05.

**Table 4:** Odds ratios of AD for *DBH *-1021TT+TC vs CC in certain subsets

Subset	Adjusted* odds ratios of AD (95% CI, *p*)		
	
	All	North Europe	North Spain
All	**1.2 (1.06-1.4, 0.005)**	**1.2 (1.05-1.4, 0.01)**	1.3 (0.97-1.7, 0.08)
Men	**1.6 (1.2-2.0, 0.0002)**	**1.7 (1.3-2.2, 0.0002)**	1.5 (0.9-2.55, 0.12)
All < 75 years	**1.6 (1.2-2.2, 0.001)**	**1.55 (1.1-2.2, 0.02)**	**1.8 (1.04-3.0, 0.03)**
Men < 75 years	**2.2 (1.4-3.3, 0.0004)**	**2.2 (1.3-3.8, 0.002)**	1.9 (0.8-4.4, 0.12)

AD = Alzheimer's disease, *DBH *= dopamine β-hydroxylase, CI = confidence interval.

* All analyses controlled for centre, age, sex and genotype of apolipoprotein E ε4.

Results in bold are significant at *p *< 0.05.

### Interactions with *IL1A* and *IL6*

We found an interaction between *DBH *-1021TT+TC and *IL1A *-889TT (Table 5): synergy factor = 1.9 (1.2-3.1, 0.005). This interaction was consistent between North Europe and North Spain. We also found a possible interaction between *DBH *-1021TT+TC and *IL6 *-174GG (Table 5), but only in North Europe: synergy factor = 1.5 (1.07-2.0, 0.02) (Table 5). We also analysed the results for *DBH *-1021TT+TC and *IL1A *-889TT when stratified by each other (Table 6). Those analyses showed that each risk factor was only associated with AD in the presence of the other factor.

**Table 5:** Interactions of *DBH *-1021TT+TC vs CC with variants of *IL1A *and *IL6 *in AD risk

Interaction with	Dataset	Numbers		Power*	Adjusted^† ^synergy factor (95% CI, *p*)
				
		Controls	AD		
*IL1A *-889TT vs TC+CC	All	6137	1535	93%	**1.9 (1.2-3.1, 0.005)**
	North Europe	5678	1078	87%	**1.7 (1.02-2.8, 0.04)**
	North Spain	459	457	32%	3.4 (0.9-12.3, 0.07)

*IL6 *-174GG vs GC+CC	All	6161	1565	95%	1.3 (0.98-1.7, 0.07)
	North Europe	5692	1084	88%	**1.5 (1.07-2.0, 0.02)**
	North Spain	469	481	44%	0.94 (0.5-1.7, 0.85)

The first column indicates the models used to represent the SNPs, *IL1A *-889T/C and *IL6 *-174G/C, in the analyses of interactions with *DBH *-1021C/T.

AD = Alzheimer's disease, *DBH *= dopamine β-hydroxylase, CI = confidence interval.

* Power to detect a synergy factor of 1.9 (first interaction) or 1.5 (second interaction) at *p *< 0.05.

^† ^All analyses controlled for centre, age, sex and genotype of apolipoprotein E ε4.

Results in bold are significant at *p *< 0.05.

**Table 6:** Odds ratios of AD for the *DBH *and *IL1A *variants*, when stratified by each other

Odds ratio of AD for:-	In the subset:-	Numbers		Adjusted^† ^odds ratio of AD (95% CI, *p*)
			
		Controls	AD	
*DBH *-1021TT+TC	*IL1A *-889TC+CC	CC: 3546	CC: 862	1.1 (0.99-1.3, 0.07)
vs CC		TT+TC: 2077	TT+TC: 516	
	*IL1A *-889TT	CC: 340	CC: 87	**2.25 (1.4-3.6, 0.0008)**
		TT+TC: 174	TT+TC: 70	

*IL1A *-889TT	*DBH *-1021CC	TC+CC: 3546	TC+CC: 862	0.95 (0.7-1.3, 0.76)
vs TC+CC		TT: 340	TT: 87	
	*DBH *-1021TT+TC	TC+CC: 2077	TC+CC: 516	**1.8 (1.3-2.6, 0.0009)**
		TT: 174	TT: 70	

AD = Alzheimer's disease, *DBH *= dopamine β-hydroxylase, *IL1A *= interleukin-1α, CI = confidence interval.

* *DBH *-1021TT+TC vs CC and *IL1A *-889TT vs TC+CC

^† ^All analyses controlled for centre, age, sex and genotype of apolipoprotein E ε4.

Results in bold are significant at *p *< 0.05.

### Other *DBH *SNPs: exon 3 Ala197Thr (rs5320), intron 10 A/G (rs1611131) and exon 11 Arg535Cys (rs6271)

There were no main effects of any of these SNPs. The overall odds ratio for 197Ala homozygotes (versus carriers of one or two copies of Thr) was 1.01 (0.8-1.25, 0.9) and for intron 10 AA (versus AG+GG) was 0.97 (0.85-1.1, 0.7). However, the interaction of 197Ala homozygotes with sex was slightly stronger than that of -1021TT+TC, but only in Northern Europeans: synergy factor = 2.3 (1.4-3.9, 0.001). The only apparently significant result for intron 10 AA was an interaction with age, only in Northern Spanish, very similar to that of -1021TT+TC: synergy factor = 2.1 (1.1-3.95, 0.025). The only apparently significant result in the preliminary analysis of Arg535Cys was probably due to chance (data not shown).

## Discussion

### Interpretation of results

We have shown a clear association between the presence of the *DBH *-1021T allele and AD (Table 4): odds ratio for -1021TT+TC versus CC = 1.2 (1.06-1.4, 0.005), controlling for centre, age, sex and *APOE *ε4 genotype. This association was nearly restricted to men < 75 years old: 2.2 (1.4-3.3, 0.0004). The interactions with sex and age were both significant (*p *= 0.01 and 0.03, respectively, Table 2). Table 3 shows that the effect of age was consistent between men and women and the effect of gender was consistent between the two age groups. All these results were consistent between North Europe and North Spain (Tables 2 &4). We therefore believe these associations to be real. However, large numbers will be needed to replicate these interactions (see the power estimates in Tables 2 &5).

We also found a probable interaction between the presence of *DBH *-1021T and *IL1A *-889TT (Table 5), thus partially replicating Mateo et al 2006 [31], who reported an interaction between *DBH *-1021TT and *IL1A *-889T. The synergy factors were consistent between North Europe and North Spain (Table 5). Also, each risk factor, i.e. *DBH *-1021T and *IL1A *-889TT, was only associated with AD risk in the presence of the interacting factor (Table 6), thus indicating epistasis. However, although the results were consistent in the three largest sample-sets, Rotterdam, Santander and OPTIMA, models for the smaller sample-sets proved unreliable. Thus we can only describe this interaction as probable, not definite. The *IL1A *-889TT genotype has been found to increase transcriptional activity in assays of promoter function [41,42]. Meta-analyses [43-45] have shown heterogeneity between studies, but a possible, weak association of the -889T allele with AD: odds ratio = 1.07 (0.99-1.16) (23 Sept 2010, 29 sample-sets: http://www.alzgene.org/).

We also found a possible interaction between *DBH *-1021T and *IL6 *-174GG, partially replicating that between *DBH *-1021TT and *IL6 *-174GG reported by Mateo et al [31]. However, in this case the interaction was only seen in North Europe and the results were inconsistent between the two European regions (Table 5) and between the seven centres. Thus, this apparent interaction may not be real. The only apparently significant results for the other two *DBH *SNPs studied in our full dataset, exon 3 Ala197Thr (rs5320) and intron 10 A/G (rs1611131), were somewhat inconsistent, precluding any firm conclusions.

The -1021T allele has consistently been associated with strikingly reduced plasma DBH activity [21,23-27]. The allele partially disrupts consensus transcriptional motifs for *n-MYC *and *MEF-2 *[26]. When *DBH *promoter/reporters were cotransfected with *n-MYC *or *MEF-2*, the allele affected the response [26]. The allele is thus functional and, although we cannot assume that it has the same effect in the brain as in the plasma, we may plausibly speculate that it does also have some influence on DBH activity in the brain. DBH catalyses the conversion of dopamine to NA. The -1021C/T SNP may therefore affect levels of both catecholamines. However, although reduced levels of NA are seen in AD brain [8-13], raised levels of dopamine have generally not been found [8,12,13]. We will therefore base the discussion below on the hypothesis that the association of the -1021T allele with AD risk is mainly due to an effect on NA levels in the brain.

### The control of inflammation in the brain

One likely result of changed DBH activity is misregulation of inflammation in the brain. The mechanisms that control inflammation in the brain differ from those in the periphery; an important part of the former control system is the noradrenergic network (reviewed in [30]). The anti-inflammatory role of NA has been shown in cultured cells and rodent brains (reviewed in [30]). Raised levels of NA reduced activation of astrocytes [46] and microglia [47-49], and lowered expression of pro-inflammatory cytokines [47-50] and chemokines [50]. Noradrenergic depletion increased production of pro-inflammatory cytokines [51] and chemokines [52], and activation of astrocytes [53] and microglia [51], and impaired microglial phagocytosis of β-amyloid [50]. Astrocytes are considered major targets of noradrenaline in the brain (reviewed in [54,55]), through their β_2_-adrenoceptors [46,54], which activate the cyclic AMP pathway [54,56], which may lead to the activation of peroxisome proliferator-activated receptors (PPARs) [56-58]. These receptors down-regulate expression of pro-inflammatory genes (PPARγ: [59]; PPARδ: [60]). The importance of the cyclic AMP pathway in AD was underlined by the recent identification of the cyclic AMP-response element-binding protein as the transcription factor of most relevance to networks of AD-related genes [61]. The inhibition of the pro-inflammatory transcription factor, nuclear factor κB, by its endogenous inhibitor, IκB, may also mediate the anti-inflammatory effects of NA [62-64]. However, the anti-inflammatory role of NA remains controversial [53] and it may even have pro-inflammatory actions in certain conditions [65-67]. Nevertheless, the predominant evidence suggests a mainly anti-inflammatory, regulatory role of NA in the brain. This role is weakened in ageing [1-3] and seriously disrupted in AD [4]. Thus, elderly non-demented carriers of the *DBH *-1021T allele with presumed low activity may be more vulnerable to low-grade inflammation in the brain. This effect has been reported to be stronger in elderly men < 80 years old [33], consistent with our results.

### Other potential mechanisms

In cell cultures and rodent brains, brain-derived neurotrophic factor (BDNF) has been reported: to be induced by NA in astrocytes and neurones [68-71]; to exert certain neuroprotective actions (reviewed in [72]); and to promote synaptic plasticity and contribute to learning and memory (reviewed in [73]). BDNF levels have been found to be decreased in the postmortem hippocampus and neocortex [74-76] in AD. A large recent meta-analysis of the *BDNF *Val66Met polymorphism [77] found that the Met allele was associated with AD in women, but not men.

Noradrenergic neurones also produce and secrete other neuromodulators and neurotrophins (reviewed in [78]). These neurones also have roles in glial energy metabolism [54,55] and the maintenance of the microvasculature [79,80] and of the blood-brain barrier [81]. NA has actions against oxidative stress [57,82,83] and against excitotoxicity [84,85]. Downstream of NA, the cyclic AMP pathway has neuroprotective and antioxidant actions in neuronal cultures [86,87]. NA protects against the neurotoxicity of β-amyloid (reviewed in [88]). However, potentially pathogenic contributions of NA to AD have also been reported [65,67,89].

## Conclusions

Our results support an association of the functional *DBH *-1021T allele with increased risk of AD in men < 75 years. Any of the above neuroprotective effects of NA (reviewed in [90]) may influence that risk and that association. However, there is considerable evidence for the role of NA in the control of inflammation in the brain (reviewed in [30]). In view therefore also of the likely interaction between *DBH *and the pro-inflammatory gene, *IL1A*, we suggest that the predominant, although not sole, mechanism of the above association with AD is misregulation of inflammation in the brain. There is substantial evidence that inflammation is an early, pre-clinical factor in the development of AD (reviewed in [91]). We have previously proposed [32] that inflammation is not only a reaction to the pathology of AD, but contributes to its onset. Our present results support that view.

## Abbreviations

AD: Alzheimer's disease; *APOE*ε4: apolipoprotein E ε4; CERAD: Consortium to Establish a Registry for Alzheimer's Disease; CI: confidence interval; CSF: cerebrospinal fluid; DBH: dopamine β-hydroxylase; *DBH*: the gene for DBH; *IL1A*: the gene for interleukin-1α; *IL6*: the gene for interleukin-6; NINCDS-ADRDA: National Institute of Neurological, Communicative Diseases and Stroke-Alzheimer's Disease and Related Diseases Association; OPTIMA: the Oxford Project to Investigate Memory and Ageing; SNP: single nucleotide polymorphism.

## Competing interests

The authors declare that they have no competing interests.

## Authors' contributions

All authors contributed to the design of the study. In addition, ADS and DJL set up the Epistasis Project, with the help of the other authors. ADS and DJL decided on the strategy of the Epistasis Project, with the help of CMvD, OC, KM, PK, RH, MC-B, DRW and EC. ADS, DJL, CMvD, OC, KM, PK, RH, MC-B, DRW and EC chose the genetic interactions to study. OC and IM produced the hypothesis for this study. KM and OB gave extensive advice on the choice of SNPs to study. DJL made the final selection of polymorphisms. HK, RB, KM, DRW, EC and IM provided DNA for genotyping. DRW gave technical advice throughout. RG and NH were responsible for the genotyping of 6 sample-sets. AA-V was responsible for the Rotterdam genotyping. MC-B and DJL decided on the analytical approach. MC-B and AO advised on statistics. DJL, MGL, MC-B and AO performed the analyses. DJL drafted the manuscript. OC submitted the manuscript and is responsible for correspondence. All authors read the manuscript, studied it critically for its intellectual content and approved the final draft.

## Pre-publication history

The pre-publication history for this paper can be accessed here:

http://www.biomedcentral.com/1471-2350/11/162/prepub

## Supplementary Material

Additional file 1 **Combarros et al 2010: The dopamine β-hydroxylase -1021C/T polymorphism is associated with the risk of Alzheimer's disease in the Epistasis Project**. Click here for file
