# Supplementary material for: The dopamine β-hydroxylase -1021C/T polymorphism is associated with the risk of Alzheimer's disease in the Epistasis Project
Source: BMC Med Genet. 2010 Nov 11;11:162. doi: 10.1186/1471-2350-11-162 (PMC2994840; doi:10.1186/1471-2350-11-162)
Supplement: Additional file 1 — Combarros et al 2010: The dopamine β-hydroxylase -1021C/T polymorphism is associated with the risk of Alzheimer's disease in the Epistasis Project. [file 1471-2350-11-162-S1.DOC]

**Combarros et al 2010: The dopamine β-hydroxylase -1021C/T polymorphism is associated with the risk of Alzheimer’s disease in the Epistasis Project**

**Additional file 1**

**Table S**1. Sample characteristics by geographical region

| Region | Subjects | Age subsets | | | Sex ratio | | *APOE*ε4 | |
| --- | --- | --- | --- | --- | --- | --- | --- | --- |
|  |  | < 75 years | > 75 years | Totals | % women | *p* (controls  vs AD) | Frequency | *p* (controls  vs AD) |
| North  Europe | Controls  AD | 2426  336 | 3342  868 | 5768  1204 | 56.8%  60.5% | 0.02 | 13.9%*  33.3% | < 0.0001 |
| North  Spain | Controls  AD | 179  182 | 347  371 | 526  553 | 67.2%  66.7% | 0.90 | 8.3%*  26.0% | < 0.0001 |
| Totals | Controls  AD | 2605  518 | 3689  1239 | 6294  1757 | 57.7%  62.4% | 0.0004 | 13.4%  31.1% | < 0.0001 |

AD = Alzheimer’s Disease.

Quality control of genotyping reduced the numbers below the above figures (see Table S3).

Fuller details, including characteristics of each of the seven sample-sets, are given in Combarros et al 2009 (J Neuroinflam 6: 22).

*Difference between North Europe and North Spain: *p* < 0.0001

**Table S**2. Ethical approval

| **Group** | **Committee** |
| --- | --- |
| Bonn | Ethics Review Board of the University of Bonn |
| Bristol | Frenchay Local Research Ethics committee, Bristol |
| Nottingham | Nottingham Research Committee 2 (NHS) |
| OPTIMA | Central Oxford Ethics Committee No 1656 |
| Oviedo | Ethical Committee of the Hospital Central de Asturias |
| Rotterdam | Medical Ethical Committee of the Erasmus MC |
| Santander | Ethical Committee of the University Hospital “Marqués de Valdecilla”, Santander |

**Table S3. Genotype distributions of the studied SNPs in controls and AD cases**

**of the seven centres**

| Gene | SNP | Centre | Controls | | | AD | | |
| --- | --- | --- | --- | --- | --- | --- | --- | --- |
| *DBH* | rs1611115  -1021C/T  rs5320  Exon 3  Ala197Thr  rs1611131  Intron 10 A/G | Bonn  Bristol  Nottingham  OPTIMA  Oviedo  Rotterdam  Santander  Totals  Bonn  Bristol  Nottingham  OPTIMA  Oviedo  Rotterdam  Santander  Totals  Bonn  Bristol  Nottingham  OPTIMA  Oviedo  Rotterdam  Santander  Totals | CC  155  33  59  150  86  3204  236  3923  Ala/Ala  191  49  82  221  114  4591  333  5581  AA  130  24  41  176  71  2488  190  3120 | CT  55  15  27  84  31  1668  117  1997  Ala/Thr  33  2  12  24  12  503  47  633  AG  78  24  44  68  28  2171  139  2552 | TT  7  1  3  9  4  238  19  281  Thr/Thr  0  0  0  1  0  16  1  18  GG  20  3  11  8  1  451  27  521 | CC  160  111  47  141  113  228  191  991  Ala/Ala  205  172  75  211  173  357  278  1471  AA  137  88  38  168  105  186  166  888 | CT  70  51  25  89  69  144  94  542  Ala/Thr  38  17  6  28  20  34  38  181  AG  93  86  51  65  61  178  106  640 | TT  7  9  3  5  7  19  28  78  Thr/Thr  3  2  0  1  0  0  1  7  GG  22  13  7  8  14  27  28  119 |
| *IL1A* | rs1800587  -889C/T  rs17561  Exon 5  Ala114Ser  rs3783550  Intron 6  A/C | Bonn  Bristol  Nottingham  OPTIMA  Oviedo  Rotterdam  Santander  Totals  Bonn  Bristol  Nottingham  OPTIMA  Oviedo  Rotterdam  Santander  Totals  Bonn  Bristol  Nottingham  OPTIMA  Oviedo  Rotterdam  Santander  Totals | CC  111  24  46  102  52  2574  220  3129  Ala/Ala  118  24  47  105  55  2574  221  3144  AA  115  25  44  115  65  2415  186  2965 | CT  78  29  38  110  50  2111  127  2543  Ala/Ser  84  29  38  115  56  2111  133  2566  AC  94  27  44  107  44  2195  167  2678 | TT  21  3  12  25  7  425  27  520  Ser/Ser  22  3  12  24  8  425  25  519  CC  22  4  6  24  7  500  39  602 | CC  123  87  36  124  95  185  162  812  Ala/Ala  129  88  39  129  100  185  183  853  AA  124  89  42  117  89  194  180  835 | CT  93  86  38  80  77  162  114  650  Ala/Ser  107  86  37  78  83  162  132  685  AC  100  86  29  95  84  162  115  671 | TT  19  25  9  29  15  44  26  167  Ser/Ser  20  25  8  29  11  44  24  161  CC  31  20  12  29  19  35  34  180 |
| *IL6* | rs1800795  -174G/C  rs2069837  Intron 2  A/G | Bonn  Bristol  Nottingham  OPTIMA  Oviedo  Rotterdam  Santander  Totals  Bonn  Bristol  Nottingham  OPTIMA  Oviedo  Rotterdam  Santander  Totals | GG  77  9  32  65  60  1824  169  2236  AA  196  22  78  207  98  4380  321  5302 | GC  95  29  41  141  51  2426  163  2946  AG  29  3  21  27  19  707  65  871 | CC  52  16  22  34  8  860  49  1041  GG  1  0  0  0  0  23  5  29 | GG  81  66  33  88  89  127  148  632  AA  202  74  72  184  146  349  279  1306 | GC  123  83  36  106  82  191  137  758  AG  30  11  11  37  19  42  54  204 | CC  37  40  15  49  19  73  48  281  GG  0  0  4  0  1  0  2  7 |

SNP = single nucleotide polymorphism, AD = Alzheimer’s disease, *DBH* = dopamine β-hydroxylase, *IL1A* = interleukin-lα, *IL6* = interleukin-6

**Table S4**. Minor allele frequencies by country

| Gene | SNP | Minor  allele | Minor allele frequencies in controls | | | |
| --- | --- | --- | --- | --- | --- | --- |
|  |  |  | Britain | Germany | The Netherlands | Spain |
| *DBH* | rs1611115  rs5320  rs1611131 | T  Thr  G | 152/762 = 19.9%  40/782 = 5.1%  180/798 = 22.6% | 69/434 = 15.9%  33/448 = 7.4%  118/456 = 25.9% | 2144/10220 = 21.0%  535/10220 = 5.2%  3073/10220 = 30.1% | 194/ 986 = 19.7%  61/1014 = 6.0%  223/ 912 = 24.5% |
| *IL1A* | rs1800587  rs17561  rs3783550 | T  Ser  C | 257/778 = 33.0%  260/794 = 32.7%  246/792 = 31.1% | 120/420 = 28.6%  128/448 = 28.6%  136/462 = 29.4% | 2961/10220 = 29.0%  2961/10220 = 29.0%  3195/10220 = 31.3% | 245/ 966 = 25.4%  255/ 996 = 25.6%  303/1016 = 29.8% |
| *IL6* | rs1800795  rs2069837 | C  G | 355/778 = 45.6%  51/716 = 7.1% | 199/448 = 44.4%  31/452 = 6.9% | 4146/10220 = 40.6%  753/10220 = 7.4% | 328/1000 = 32.8%  94/1016 = 9.3% |

SNP = single nucleotide polymorphism, *DBH* = dopamine β-hydroxylase, *IL1A* = interleukin-lα, *IL6* = interleukin-6

Combarros2010,DBH,AddtllTables,8Nov’10
